# Incidence, Antimicrobial Susceptibility, and Toxin Genes Possession Screening of *Staphylococcus aureus* in Retail Chicken Livers and Gizzards

**DOI:** 10.3390/foods4020115

**Published:** 2015-04-21

**Authors:** Lubna S. Abdalrahman, Mohamed K. Fakhr

**Affiliations:** Department of Biological Science, The University of Tulsa, Tulsa, OK 74104, USA; E-Mail: Lubna-abdalrahman@utulsa.edu

**Keywords:** *Staphylococcus aureus*, antibiotic resistance, toxins, toxin genes, prevalence, chicken livers, chicken gizzards, antimicrobials, foodborne pathogens, retail meat

## Abstract

Few recent outbreaks in Europe and the US involving *Campylobacter* and *Salmonella* were linked to the consumption of chicken livers. Studies investigating *Staphylococcus aureus* in chicken livers and gizzards are very limited. The objectives of this study were to determine the prevalence, antimicrobial resistance, and virulence of *S. aureus* and MRSA (Methicillin-Resistant *Staphylococcus aureus*) in retail chicken livers and gizzards in Tulsa, Oklahoma. In this study, 156 chicken livers and 39 chicken gizzards samples of two brands were collected. While one of the brands showed very low prevalence of 1% (1/100) for *S. aureus* in chicken livers and gizzards, the second brand showed prevalence of 37% (31/95). No MRSA was detected since none harbored the *mecA* or *mecC* gene. Eighty seven *S. aureus* isolates from livers and 28 from gizzards were screened for antimicrobial resistance to 16 antimicrobials and the possession of 18 toxin genes. Resistance to most of the antimicrobials screened including cefoxitin and oxacillin was higher in the chicken gizzards isolates. While the prevalence of enterotoxin genes *seg* and *sei* was higher in the gizzards isolates, the prevalence of hemolysin genes *hla*, *hlb*, and *hld* was higher in the livers ones. The lucocidin genes *lukE-lukD* was equally prevalent in chicken livers and gizzards isolates. Using *spa* typing, a subset of the recovered isolates showed that they are not known to be livestock associated and, hence, may be of a human origin. In conclusion, this study stresses the importance of thorough cooking of chicken livers and gizzards since it might contain multidrug resistant enterotoxigenic *S. aureus*. To our knowledge this is the first study to specifically investigate the prevalence of *S. aureus* in chicken livers and gizzards in the US.

## 1. Introduction

Recent studies highlighted the importance of chicken livers as a food safety hazard. A US study found that 77% of retail chicken livers and 33% of chicken gizzards were contaminated with *Campylobacter* [1]. Several recent outbreaks involving *Campylobacter* from poultry livers have occurred in Europe [2,3,4,5] and in the US [6,7]. A US outbreak of *Salmonella* Heidelberg was linked to Kosher Broiled Chicken Livers [8]. One study—as a part of a larger study on retail raw chicken meat throughout Japan—reported a high prevalence of *S. aureus* in chicken livers (63.8%) and chicken gizzards (58.1%) after enrichment [9]. Another study in Turkey reported 9/30 of chicken giblets (30%) to be positive for the bacterium [10].

*Staphylococcus aureus* is the fifth pathogen causing domestically acquired foodborne illnesses annually in the United States [11] and its food poisoning is an important cause of food-borne diseases worldwide [12]. Several staphylococcal enterotoxins and enterotoxin-like superantigens have been described [13,14,15]. Staphylococcal food poisoning is an intoxication resulting from the consumption of food that has been contaminated with the toxin itself and most of its symptoms are self-limiting within one to two days after consumption [16]. *Staphylococcus aureus* has the ability to destroy red blood cells by producing three types of hemolysins, known as alpha, beta and delta toxins [17]. The beta hemolysin gene is considered dangerous since it encodes the beta toxins that have the ability to inhibit the ciliary activity of human lungs and cornea [18,19,20]. 

Most studies in the literature specifically reporting prevalence of MRSA (Methicillin-Resistant *Staphylococcus aureus*) in food animals were conducted in pigs [21,22,23,24]. *S. aureus* and MRSA have been also isolated and was highly prevalent in poultry in the Netherlands [25]. It was also detected in retail chicken meat in Japan [9]. In the last few years, few studies were conducted to determine the prevalence of *S. aureus* and MRSA in retail meats in several states in the United States that includes North Dakota [26,27], Georgia [28], Minnesota, and New Jersey [29], Iowa [30], Detroit, Michigan [31], Maryland [32] and Louisiana, [33]. Only few of those studies included data from poultry such as ground turkey [32], chicken and/or turkey [26,27,30,31]. While most studies conducted in Europe identified MRSA isolated from retail meats as belonging to Livestock associated MRSA strains (LA-MRSA), the majority of MRSA isolates detected in the US retail meats were human associated strains except few that were LA-MRSA and were mostly reported in pork meat. So, opposite to the case in Europe, US retail meats when contaminated with MRSA the origin is most likely from human handling the meat products.

Studies investigating the prevalence of *S. aureus* and MRSA in chicken livers and gizzards are very limited [9] and to our knowledge none is available to date from the US. Chicken livers in particular are usually undercooked to preserve taste and texture which can be risky if contaminated with enterotoxin producing strains of *S. aureus* specially that most of these enterotoxins are heat stable. The objectives of this study were to determine the prevalence of *S. aureus* and MRSA in retail chicken livers and gizzards collected in Tulsa, Oklahoma and to characterize the recovered isolates for their antimicrobial susceptibility and possession of toxin genes. To our knowledge this study is the first to specifically investigate the prevalence of *S. aureus* in chicken livers and gizzards in the US.

## 2. Experimental Section 

### 2.1. Isolation of Staphylococcus aureus from Retail Chicken Livers and Gizzards

Chicken livers and gizzards samples were collected from several different grocery stores in the Tulsa, Oklahoma area for a period of six months starting January of 2010. A total of 195 chilled retail chicken liver and chicken gizzard samples were used in this study (156 chicken livers and 39 chicken Gizzards) (Table 1). Meat samples were purchased from nine grocery stores that belong to six different franchises chains at variable locations in the city. The chicken livers and gizzards belonged to two major brands, which are designated brand A and brand B (Table 1). Samples were selected to be as variable as possible with different expiration and production dates. Chicken livers and gizzards samples were added to 10 mL of buffered peptone water (BPW) (BPW; EMD, Gibbstown, NJ, USA) in sterile plastic bags (VWR Scientific, Radnor, PA, USA) and massaged by hand for approximately 5 min. Ten milliliters was then transferred from the bag and added to 10 mL of enrichment broth of 2× Trypticase Soy Broth with 10% sodium chloride and 1% sodium pyruvate, then incubated at 37 °C for 24 h. A loopful was then streaked to Baird Parker (BP) selective media plates and incubated at 37 °C for 48 h [33]. Four suspected *S. aureus* colonies (those that have black colonies surrounded by 2 to 5 mm clear zones) were selected and streaked to Trypticase Soy Agar (TSA) plates and subcultured for confirmation on MSA (Mannitol Salt Agar) plates. Pure prospective *S. aureus* cultures were kept at −80 °C until PCR confirmation.

**Table 1:** PCR primers and their references for *Staphylococcus aureus* and MRSA identification.

Gene	Size (bp)	Primer sequences (5´–3´)	Bacterium	References
Sa4221-1	108	AAT CTT TGT CGG TAC ACG ATA TTC TTC ACG	*S. aureus*	[34]
Sa4221-2		CGT AAT GAG ATT TCA GTA GAT AAT ACA ACA		
*mecA-F*	312	GTT GTA GTT GTC GGG TTT GGCTT CCA CAT ACC ATC TTC TTT AAC	*MRSA*	[21]
*mecA-R*		CTT CCA CAT ACC ATC TTC TTT AAC		
*mecA1F*	533	AAA ATC GAT GGT AAA GGT TGG C	*MRSA*	[35]
*mecA2R*		AGT TCT GCA GTA CCG GAT TTG C		
*MecHomFW*	356	TCA CCA GGT TCA AC[Y] CAA AA	*MRSA*	[36]
*MecHomRV*		CCT GAA TC[W] GCT AAT AAT ATT TC		
*mecAM10/0061 F1*	1800	CCA GAT ATA GTA GCA TTA TA	*MRSA*	[37]
*mecAM10/0061 R1*		AAA GAT GAC GAT ATT GAG		

### 2.2. DNA Extraction

DNA was extracted from the prospective *S. aureus* strains using the single cell lysing buffer (SCLB) method [38]. One day old colonies were picked and suspended in 40 μL of single cell lysing buffer (SCLB) solution (1.0 mL of TE buffer (10 mM Tris-HCL and 1 mM EDTA) and 10 μl of 5 mg/mL proteinase K) in a 0.2 mL microtube. In a thermocycler, bacterial cells were lysed by initial incubation at 80 °C for 10 min, followed by 55 °C for 10 min, and then 95 °C for 10 min [38]. DNA extracted by the above mentioned method was stored at −20 °C until used as a DNA template for PCR. 

### 2.3. PCR Identification

A multiplex PCR reaction was used to identify the isolated suspected *S. aureus* by using specific primers for *S. aureus* and MRSA to amplify a 108 bp and a 312 bp fragments respectively (Table 4)*.* Twenty microliters PCR reactions, which included 10 µL of Qiagen Master Mix (Qiagen Inc., Valencia, CA, USA), 4 µL of sterile water (Qiagen), 1 µL of each forward and reverse primer (IDT, Coralville, IA, USA), and 2 µL of DNA template, were performed. The PCR protocol was as follows: initial denaturing at 95 °C for 5 min (followed by 35 cycles of denaturing at 95 °C for 1 min, annealing at 55 °C for 1 min, and extension at 72 °C for 1 min) and ending with extension at 72 °C for 10 min. PCR amplicons were subjected to agarose gel electrophoresis and DNA bands were visualized and recorded using a gel documentation system. Isolates showing resistance to cefoxitin and/oxacillin were subjected to PCR confirmation using a second set of MRSA primers that amplify a 533 bp *mecA* fragment and two other variant MRSA *mecA* primer sets (also known as *mecC*) that amplify 356 bp and 1800 bp fragments to confirm the MRSA phenotype (Table 1).

### 2.4. Antimicrobial Susceptibility Testing

A total of 115 *S. aureus* recovered isolates (87 chicken liver isolates and 28 chicken gizzard isolates) were subjected to antimicrobial resistance profiling against sixteen different antimicrobials that belong to ten different antibiotic classes (Table 2). Isolates were grown on Mueller-Hinton (MH) agar (Difco) and incubated for 48 h at 37 °C. Cultures were then added to Mueller-Hinton broth (Difco), adjusted to turbidity equal to a 0.5 McFarland standard, and inoculated onto 6-inch MH agar plates supplemented with the appropriate antimicrobial at different concentrations (Table 2) including the breakpoint established for each antimicrobial according to the Clinical and Laboratory Standards Institute (CLSI) when available [39]. Plates were then incubated at 37 °C for 48 h and results were read for growth or no growth and denoted as resistant or susceptible, respectively according to the breakpoints for each of the tested antimicrobials (Table 2).

**Table 2:** A list of the 16 antimicrobials, their classes, concentrations used for susceptibility testing, and the breakpoints used for each antimicrobial.

Antimicrobial Class	Antimicrobials	Conc. 1 * (µg/mL)	Conc. 2 (µg/mL) (Break point)	Conc. 3 (µg/mL)	Conc. 4 (µg/mL)
β-Lactams	penicillin	0.125	0.25	0.5	1
	ampicillin	0.25	0.5	1	2
	oxacillin + 2% Nacl	2	4	8	16
	cefoxitin + 2% Nacl	4	8	16	32
Tetracyclines	tetracycline	8	16	32	64
	doxycycline	8	16	32	64
Macrolides	azithromycin	4	8	16	32
	erythromycin	4	8	16	32
Aminoglycosides	kanamycin	32	64	128	256
	gentamicin	8	16	32	64
Fluoroquinolones	ciprofloxacin	2	4	8	16
Lincosamides	clindamycin	2	4	8	16
Phenicols	chloramphenicol	16	32	64	128
Glycopeptides	vancomycin	16	32	64	128
Rifamycines	rifampin	2	4	8	16
Sulfonamides	trimethoprim/sulfamethoxazole	2/38	4/76	8/152	16/304

* Conc.: Concentration.

### 2.5. Detection of Toxin Genes

A total of 115 *Staphylococcus aureus* isolates (87 chicken liver isolates and 28 chicken gizzard isolates) were screened for eighteen different toxin genes that belong to six different toxin gene groups (Table 3). Multiplex PCR was used to detect 18 different toxin genes of *S. aureus* isolates that include enterotoxins, toxic shock syndrome toxin 1, exfoliative toxins, leucocidins, Panton-Valentine leucocidin (PVL), and hemolysins (Table 3). Three multiplex reactions (A, B, and C), each of which included six toxin genes, were performed (Table 3). The multiplex PCR targeting the toxin genes were performed in a 20 µL reaction solution that contained 10 µL of Green Master Mix (Promega), 2µL of sterile water, 2 µL of the DNA template and 0.5 µL of each of the toxin gene primers. The PCR protocol included an initial denaturation at 95 °C for 5 min, followed by 30 cycles of denaturation (94 °C for 1 min), annealing (57 °C for 1 min), and extension (72 °C for 1 min), ending with an extension at 72 °C for 7 min. PCR amplicons were subjected to agarose gel electrophoresis and DNA bands were visualized and recorded using a gel documentation system. The expected amplicon band sizes of *S. aureus* toxin genes are shown in Table 3. Several representative amplicons of each positive toxin were sequenced in house using the same amplifying primers to confirm PCR accuracy.

**Table 3:** Multiplex PCR primers, reaction sets, references, and toxin groups for the screened toxin genes.

Toxin Gene (Toxin group)	Size (bp)	Primer sequences (5´–3´)	Multiplex PCR reaction set	References
*sea* (Enterotoxins)	521	GCA GGG AAC AGC TTT AGG C GTT CTG TAG AAG TAT GAA ACA CG	A	[40]
*seb-sec* (Enterotoxins)	665	ATG TAA TTT TGA TAT TCG CAG TG TGC AGG CAT CAT ATC ATA CCA	A	[40]
*sec* (Enterotoxins)	284	CTT GTA TGT ATG GAG GAA TAA CAA TGC AGG CAT CAT ATC ATA CCA	A	[40]
*sed* (Enterotoxins)	385	GTG GTG AAA TAG ATA GGA CTG C ATA TGA AGG TGC TCT GTG G	A	[40]
*see* (Enterotoxins)	171	TAC CAA TTA ACT TGT GGA TAG AC CTC TTT GCA CCT TAC CGC	A	[40]
*seg* (Enterotoxins)	328	CGT CTC CAC CTG TTG AAG G CCA AGT GAT TGT CTA TTG TCG	A	[40]
*seh* (Enterotoxins)	359	CAA CTG CTG ATT TAG CTC AG GTC GAA TGA GTA ATC TCT AGG	B	[40]
*sei* (Enterotoxins)	466	CAA CTC GAA TTT TCA ACA GGT AC CAG GCA GTC CAT CTC CTG	B	[40]
*sej* (Enterotoxins)	142	CAT CAG AAC TGT TGT TCC GCT AG CTG AAT TTT ACC ATC AAA GGT AC	B	[40]
*tst* (Toxic Shock Syndrome Toxin 1)	560	GCT TGC GAC AAC TGC TAC AG TGG ATC CGT CAT TCA TTG TTA A	B	[40]
*eta* (Exfoliative toxins)	93	GCA GGT GTT GAT TTA GCA TT AGA TGT CCC TAT TTT TGC TG	B	[41]
*etb* (Exfoliative toxins)	226	ACA AGC AAA AGA ATA CAG CG GTT TTT GGC TGC TTC TCT TG	B	[41]
*lukS-lukF* (Panton-Valentine leucocidin (PVL))	433	ATC ATT AGG TAA AAT GTC TGG ACA TGA TCC A GCA TCA AST GTA TTG GAT AGC AAA AGC	C	[42]
*lukE-lukD* (Leucocidin)	269	TGA AAA AGG TTC AAA GTT GAT ACG AG TGT ATT CGA TAG CAA AAG CAG TGC A	C	[42]
*lukM* (Leucocidin)	780	TGG ATG TTA CCT ATG CAA CCT AC GTT CGT TTC CAT ATA ATG AAT CAC TAC	C	[42]
*hla* (Hemolysins)	209	CTG ATT ACT ATC CAA GAA ATT CGA TTG CTT TCC AGC CTA CTT TTT TAT CAG T	C	[42]
*hlb* (Hemolysins)	309	GTG CAC TTA CTG ACA ATA GTG C GTT GAT GAG TAG CTA CCT TCA GT	C	[42]
*hld* (Hemolysins)	111	AAG AAT TTT TAT CTT AAT TAA GGA AGG AGT G TTA GTG AAT TTG TTC ACT GTG TCG A	C	[42]

### 2.6. Molecular Typing Using spa Genotyping

A subset of the recovered *Staphylococcus aureus* isolates were subjected to molecular typing using *spa* typing. Molecular typing using *spa* was done according to published primers and protocols [43] and *spa* types were assigned using the BioNumerics Software (Applied Math, Austin, TX, USA) through the Ridom Spa Server. 

## 3. Results and Discussion

### 3.1. Prevalence of Staphylococcus aureus and MRSA in Chicken Livers and Gizzards

A total of 195 chilled retail chicken liver and chicken gizzard samples were purchased from several Tulsa area grocery stores starting January 2010 for a period of 6 months. The number of chicken liver samples was 156 and the number of chicken gizzard samples was 39 (Table 1). The chicken livers and gizzards collected in this study belonged to two major brands, which were designated brand A and brand B (Table 4). As shown in Table 4, the overall prevalence of *S. aureus* in chicken livers and gizzards including the two brands together was 36/195 (18.5%). While 27/156 (17.3%) of chicken livers were contaminated with *S. aureus*, 9/39 (23.1%) of chicken gizzards were positive for the bacterium. The prevalence of *S. aureus* in brand A chicken livers was 26/71 (36.6%) while it was 9/24 (37.5%) in chicken gizzards of the same brand (Table 1). Only one out of 85 chicken liver samples (1.2%) of brand B was positive for *S. aureus* and none of the chicken gizzards of this brand wa*s* positive for *S. aureus*. No isolates of chicken livers and gizzards were positive for MRSA since none of them carried *mecA* or *mecC* genes.

Even though the overall prevalence of *S.* *aureus* in chicken livers and gizzards was 36/195 (18.5%) in our study, the 36.6% and the 37.5% prevalence in brand A chicken livers and gizzards respectively is alarming (Table 4). While there were no available studies in the literature that specifically determined the prevalence of *S.* *aureus* in chicken livers and gizzards, a study in Turkey reported contamination in 9/30 (30%) of chicken giblets as a part of a larger study on chicken meat [10]. A second study in Japan reported a higher prevalence of *S. aureus* in chicken livers (63.8%) and chicken gizzards (58.1%) after enrichment while it was 47.9% and 22.6% respectively before enrichment [9]. The higher prevalence in the Japanese study might be due to differences between the US and the Japanese retail poultry markets. It can also be due to the methods used for identification since the Japanese study used only biochemical tests for identification of the *S. aureus* strains while molecular identification was used in our study. *S. aureus* was isolated from 56% of ground turkey collected from Maryland, USA [32] and was found in 25% of retail chicken in Detroit, Michigan where 3.9% were MRSA [31]. In another study in Iowa, 17.8% of retail chicken was contaminated with *S. aureus* [30], while in North Dakota a higher prevalence of *S. aureus* (67.6%) in retail chicken was reported [26].

The big difference between the overall prevalence of *S. aureus* in chicken livers and gizzards in brand A in our study (36.8%) and only 1% in brand B (Table 4) might be due to the difference in food safety and microbiology quality control handling protocols at the two production companies. While not conclusive, this data might suggest that chicken livers and gizzards contamination with *S. aureus* most probably occurs during handling at the slaughter house or at the retail packaging step.

**Table 4:** Prevalence of *Staphylococcus aureus* in the 195 collected chicken liver and gizzards samples.

Prevalence of *Staphylococcus aureus*								
Chicken Livers			Chicken Gizzards			Chicken Livers and Gizzards		
Brand A *np/n* * (%)	Brand B *np/n* (%)	Total *np/n* (%)	Brand A *np/n* (%)	Brand B *np/n* (%)	Total *np/n* (%)	Brand A *np/n* (%)	Brand B *np/n* (%)	Total *np/n* (%)
26/71 (36.6)	1/85 (1.2)	27/156 (17.3)	9/24 (37.5)	0/15 (0)	9/39 (23.1)	35/95 (36.8)	1/100 (1)	36/195 (18.5)

* *np*: number of positive samples, *n*: number of samples collected.

### 3.2. Antimicrobial Susceptibility of the Recovered Isolates

A total of 115 *Staphylococcus aureus* isolates (87 chicken liver isolates and 28 chicken gizzard isolates) were subjected to antimicrobial resistance profiling against sixteen different antimicrobials that belong to ten different antibiotic classes (Table 2 and Table 5). As shown in Table 5, the percentage of resistance of the 115 *S. aureus* isolates from chicken livers and gizzards to the sixteen tested antimicrobials were as follow: ampicillin (88.9%), tetracycline (71.3%), doxycycline (63.5%), penicillin (60.9%), erythromycin (45.2%), azithromycin (40,9%), vancomycin (39.1%), oxacillin with 2% NaCl (32.2%), ciprofloxacin (29.6%), trimethoprim/sulfamethazole (24.3%), rifampin (23.5%), cefoxitin with 2% NaCl (19.1%), clindamycin (12.2%), kanamycin (12.2%), chloramphenicol (10.4%), and gentamicin (10.4%). As shown in Table 5, the percentage of resistance to the sixteen tested antimicrobials varied between chicken livers and chicken gizzards isolates. The percentage of resistance found in the chicken gizzards was higher than chicken livers isolates for the following 10 antimicrobials: azithromycin, ciprofloxacin, oxacillin, cefoxitin, tetracycline, vancomycin, doxycycline, penicillin, kanamycin, and erythromycin (Table 5). On the other hand, for gentamycin, ampicillin, trimethoprim/sulfamrthoxazole, clindamycin, rifampin, chloramphenicol the chicken livers isolates showed a higher resistance (Table 5). This variability in antimicrobial resistance between isolates from chicken livers and gizzards might be attributed to the concentration of different antimicrobials in the liver and/or the fact that chicken gizzards often have more fats that would make some highly lipid soluble antimicrobials like azithromycin get to higher concentrations in the gizzards. Overall 35/115 (30%) of the screened isolates from chicken livers and gizzards were multidrug resistant to more than seven antimicrobials (data not shown) which is worrisome.

Resistance to vancomycin was relatively high in isolates from chicken livers and gizzards in our study (Table 5).Vancomycin Resistant *Enterococcus faecium* (VRE) was previously reported in swine in Michigan, USA and was thought to be widespread despite the historical absence of the use of agricultural glycopeptides like avoparcin. Screening our phenotypically vancomycin strains for the presence of the *vanA* gene is currently underway as a part of a larger study focusing on vancomycin resistant *S. aureus* strains isolated from various US retail meats. Chicken livers and gizzards isolates in our study were highly resistant to ampicillin, tetracycline, doxycycline, penicillin, and erythromycin (Table 5). The literature is lacking data about antimicrobial resistance of *S. aureus* strains isolated from chicken livers and gizzards. One study in Turkey [10] reported that *S. aureus* isolates from chicken giblets were resistant to penicillin G (22.2%) and erythromycin (33.3%). *S. aureus* recovered strains in our study that showed resistance to cefoxitin and/or oxacillin (highly prevalent in chicken gizzards as shown in Table 5) were subjected to additional PCR protocols to check for the presence of a *mecA* homologue (Table 1). None of these isolates showed the presence of *mecA* gene or it homologues (*mecC*). Phenotypic MRSA isolates that do not harbor the *mecA* gene were reported [32,44]. This might be due to over production of Beta-lactamase enzymes or the presence of a variant *mecA* gene that does not amplify with the known PCR primers. The recent advancement in whole genome sequencing through next generation sequencing can help identifying such homologues. 

The high number of multidrug resistant *S. aureus* detected in our study is alarming. It raises concerns about inappropriate practices including the use of antimicrobials as growth promotors in food animal production and the frequent use of antimicrobials in poultry husbandry. Genes coding for antimicrobial resistance can move through horizontal gene transfer to clinical pathogenic strains and contribute to the creation of superbugs. Death in hospitals are often attributed to sepsis resulting from infections caused by multidrug resistant pathogens like MRSA, *Pseudomonas aeruginosa* or *Candida albicans* rather than the original cause of the hospitalization. 

**Table 5:** Antimicrobial resistance of the 115 *Staphylococcus aureus* chicken liver and gizzard isolates against 16 different antimicrobials.

Antimicrobial Resistance			
Antibiotic	Chicken Livers *np*/*n* (%) *	Chicken Gizzards *np*/*n* (%)	Chicken Livers and Gizzard *np*/*n* (%)
azithromycin	25/87 (28.7)	22/28 (78.6)	47/115 (40.9)
ciprofloxacin	13/87 (14.9)	21/28 (75.0)	34/115 (29.6)
gentamicin	10/87 (11.5)	2/28 (7.1)	12/115 (10.4)
oxacillin	19/87 (21.8)	18/28 (64.3)	37/115 (32.2)
cefoxitin	10/87 (11.5)	12/28 (42.9)	22/115 (19.1)
tetracycline	59/87 (67.8)	23/28 (82.1)	82/115 (71.3)
vancomycin	30/87 (34.5)	15/28 (53.6)	45/115 (39.1)
doxycycline	49/87 (56.3)	24/28 (85.7)	73/115 (63.5)
trimethoprim/sulfamethoxazole	22/87 (25.3)	6/28 (21.4)	28/115 (24.3)
clindamycin	13/87 (14.9)	1/28 (3.6)	14/115 (12.2)
penicillin	47/87 (54.0)	23/28 (82.1)	70/115 (60.9)
ampicillin	83/87 (95.4)	19/28 (67.9)	102/115 (88.9)
kanamycin	17/87 (19.5)	7/28 (25.0)	14/115 (12.2)
erythromycin	31/87 (35.6)	21/28 (75.0)	52/115 (45.2)
rifampin	25/87 (28.7)	2/28 (7.1)	27/115 (23.5)
chloramphenicol	11/87 (12.6)	1/28 (3.6)	12/115 (10.4)

* *np*: number of positive isolates, *n*: number of isolates collected.

### 3.3. Toxin Genes Possession Screening of the Recovered Isolates

A total of 115 *Staphylococcus aureus* isolates (87 chicken liver isolates and 28 chicken gizzard isolates) were screened for eighteen different toxin genes that belong to six different toxin gene groups (Tables 3 and 6). As shown in Table 6, the prevalence of toxin genes in the 115 *S. aureus* isolates from chicken livers and gizzards to the eighteen tested toxin genes were as follow: *hla* (94.5%), *hld* (94.5%), *hlb* (48.7%), *sei* (42.6%), *lukE-lukD* (36.5%), *seg* (29.6%), *seh* (4.3%), *sed* (0.9%), *sea* (0%), *seb-sec* (0%), *sec* (0%), *see* (0%), *sej* (0%), *tst* (0%), *eta* (0%), *etb* (0%), *lukM* (0%), and *lukS-lukF* (0%). *S. aureus* hemolysin genes were found at a higher percentage in the chicken livers and gizzards than other groups of toxin genes screened. Also no isolates harbored enterotoxin genes *sed*, *sea*, *seb-sec*, *sec*, *see*, or *sej*, the toxic shock syndrome toxin 1 gene *tst*, the exfoliative toxin genes *eta*, *etb*, or the Leucocidin gene *lukM* (Table 6). The prevalence of enterotoxin genes *seg* (71.43%) and *sei* (92.9%) in chicken gizzards was higher than in chicken livers, where *seg* was 16.1% and *sei* was 26.4%. One isolate from chicken livers was positive for the entoretoxin gene *sed* (1.2%) and 23/87 of chicken livers isolates were positive for the entoretoxin gene *sei* (26.4%). The prevalence of hemolysin genes *hla* (97.5%), *hld* (97.5%) and *hlb* (64.4%) in chicken livers was higher than in chicken gizzards when it was 85.7%, 85.7% and 0% respectively. The Hemolysin gene *hlb* was present only in the chicken livers. Both chicken livers and gizzards isolates had similar prevalence for *lukE-lukD*. 

**Table 6:** The prevalence of 18 different toxin genes in the 115 *Staphylococcus aureus* chicken liver and gizzard isolates.

Prevalence of Toxin Genes			
Toxin Gene	Chicken Livers *np*/*n* (%) *	Chicken Gizzards *np*/*n* (%)	Chicken Livers and Gizzards *np*/*n* (%)
*sea*	0/87 (0)	0/28 (0)	0/115 (0)
*seb-sec*	0/87 (0)	0/28 (0)	0/115 (0)
*sec*	0/87 (0)	0/28 (0)	0/115 (0)
*sed*	1/87 (1.2)	0/28 (0)	1/115 (0.9)
*see*	0/87 (0)	0/28 (0)	0/115 (0)
*seg*	14/87 (16.1)	20/28 (71.43)	34/115 (29.6)
*seh*	5/87 (5.8)	0/28 (0)	5/115 (4.3)
*sei*	23/87 (26.4)	26/28 (92.9)	49/115 (42.6)
*sej*	0/87 (0)	0/28 (0)	0/115 (0)
*tst*	0/87 (0)	0/28 (0)	0/115 (0)
*eta*	0/87 (0)	0/28 (0)	0/115 (0)
*etb*	0/87 (0)	0/28 (0)	0/115 (0)
*lukE-lukD*	32/87 (36.8)	10/28 (35.7)	42/115 (36.5)
*lukM*	0/87 (0)	0/28 (0)	0/115 (0)
*hla*	85/87 (97.7)	24/28 (85.7)	109/115 (94.5)
*hlb*	56/87 (64.4)	0/28 (0)	56/115 (48.7)
*hld*	85/87 (97.7)	24/28 (85.7)	109/115 (94.5)
*lukS-lukF*	0/87 (0)	0/28 (0)	0/115 (0%)

* *np*: number of positive isolates, *n*: number of isolates collected.

The literature is lacking data related to the prevalence of toxin genes in *S. aureus* isolated from chicken livers and gizzards. Even recent studies discussing *S. aureus* in US retail poultry in general lacks such toxin genes prevalence data. A study in Japan reported that 25.3% of their chicken liver isolates were enterotoxigenic while 36.4% of their chicken gizzards produced enterotoxins [9]. Even though they have not used PCR to detect enterotoxin genes in the Japanese study, their chicken gizzards strains showed a higher prevalence of enterotoxins that their chicken livers ones which is in agreement with our findings. The higher prevalence of hemolysin genes in chicken livers isolates than in the chicken gizzards ones might be due to the availability of blood in the liver which might select for *S. aureus* strains with blood lysing abilities. Chicken livers and gizzards should be cooked thoroughly since enterotoxins of *S. aureus* are known for their heat tolerance. So even if the cooking temperature was high enough to kill the pathogen, enterotoxins produced on the chicken livers or gizzards could tolerate such temperature increasing the risk of food poisoning.

### 3.4. Genotyping Using spa Typing 

**Figure 1:**
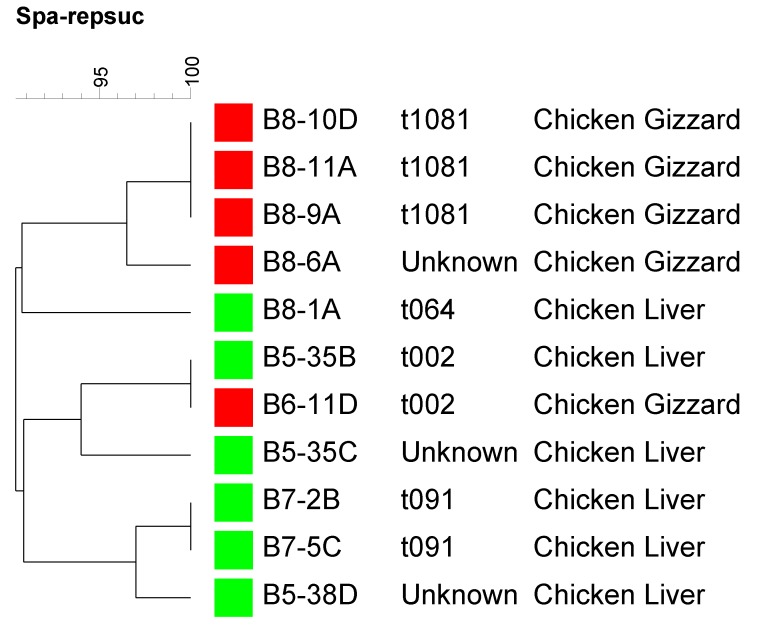
A dendrogram showing *spa* typing for a subset of the recovered *Staphylococcus aureus* strains representing chicken livers and chicken gizzards sources. Strains isolated from the same meat source are labeled by the same color square.

A subset of *Staphylococcus aureus* recovered strains (6 from chicken livers, and 5 from chicken gizzards) were subjected to molecular typing by *spa* typing (Figure 1). As it is shown in Figure 1, *spa* types were grouped into two major clusters with the majority of isolates in each cluster belonging to one source. As it is also shown in figure 1, the tested isolates showed higher diversity in regards to their *spa* types since 7 different *spa* types were detected among a subset of 11 isolates. The detected *spa* types (t1081, t064, t002, and t091) were not known to be livestock associated and hence, maybe of a human origin [45]. This is in agreement with what we discussed earlier in the introduction section about that the origin of *S. aureus* strains detected in US retail meats is mostly of a human origin rather than livestock associated as it is the case in Europe. 

## 4. Conclusions

The prevalence of *S. aureus* in retail chicken livers and gizzards tested in this study varied between the two brands tested. While one of the brands showed very low prevalence of *S. aureus*, the second brand showed prevalence close to 37%. The percentage of resistance to most of the antimicrobials screened was generally higher in isolates recovered from chicken gizzards. While no isolate harbored the *mecA* or *mecC* gene, a higher percentage of the chicken gizzards isolates were resistant to cefoxitin and/or oxacillin making them phenotypically similar to MRSA. A high percentage of *S. aureus* recovered strains particularly from chicken gizzards harbored enterotoxins *seg* and *sei*. The lucocidin genes *lukE-lukD* was equally prevalent in chicken livers and gizzards isolates. The hemolysin *hlb* gene was only prevalent in the chicken livers strains while *hla* and *hld* were prevalent in chicken livers and gizzards strains. Using *spa* typing, a subset of the recovered isolates showed that they are not known to be livestock associated and hence, maybe of a human origin. Data obtained from this study stress the importance of thorough cooking of chicken livers and gizzards since it might contain multidrug resistant enterotoxigenic *S. aureus*. 
